# Ratio abstraction over discrete magnitudes by newly hatched domestic chicks (*Gallus gallus*)

**DOI:** 10.1038/srep30114

**Published:** 2016-07-28

**Authors:** Rosa Rugani, Koleen McCrink, Maria-Dolores de Hevia, Giorgio Vallortigara, Lucia Regolin

**Affiliations:** 1 Department of General Psychology, University of Padova, Padova, Italy; 2 Department of Psychology, Barnard College, Columbia University, USA; 3 Université Paris Descartes, Sorbonne Paris Cité, Paris, France; 4 CNRS UMR 8242, Laboratoire Psychologie de la Perception, Paris, France; 5 Center for Mind/Brain Sciences, University of Trento, Rovereto (Trento), Italy

## Abstract

A large body of literature shows that non-human animals master a variety of numerical tasks, but studies involving proportional discrimination are sparse and primarily done with mature animals. Here we trained 4-day-old domestic chicks (*Gallus gallus*) to respond to stimuli depicting multiple examples of the proportion 4:1 when compared with the proportion 2:1. Stimuli were composed of green and red dot arrays; for the rewarded 4:1 proportion, 4 green dots for every red dot (e.g. ratios: 32:8, 12:3, and 44:11). The birds continued to discriminate when presented with new ratios at test (such as 20:5), characterized by new numbers of dots and new spatial configurations (Experiment 1). This indicates that chicks can extract the common proportional value shared by different ratios and apply it to new ones. In Experiment 2, chicks identified a specific proportion (2:1) from either a smaller (4:1) or a larger one (1:1), demonstrating an ability to represent the specific, and not relative, value of a particular proportion. Again, at test, chicks selectively responded to the previously reinforced proportion from new ratios. These findings provide strong evidence for very young animals’ ability to extract, identify, and productively use proportion information across a range of different amounts.

Humans and non-human animals can solve rudimentary mathematical operations such as ordering, addition and subtraction over discrete numbers^1^,^2^,^3^,^4^,^5^,^6^,^7^; for a review see ref. 8), but studies involving discrimination of numerical ratios over discrete amounts are scarce. Some theorists have posited that the representational format of proportional information is comparable to the representation of discrete numbers^9^,^10^,^11^, which suggests that proportional reasoning would also be present in those same young animal populations who can represent discrete number. For this reason, here we use very young domestic chicks as a test case of whether immature organisms can extract proportional information over discrete sets of objects, without a significant amount of life experience. If this proportional information is represented in a specific, non-relative, fashion, these naïve newborn chicks will be able to flexibly compare proportions to other proportional magnitudes that are larger or smaller.

Several studies have shown prowess with discrete numerical magnitude in mature birds^12^,^13^,^14^ as well as newborn chicks^15^,^16^,^17^,^18^. For example, Emmerton and Renner^12^ trained pigeons to respond to a particular “anchor” amount, and found that the birds were able to select this numerical magnitude in the context of multiple comparisons, and despite changes in surface features such as area or luminance. These representations are thought to be supported by an evolutionarily ancient system for representing magnitudes in an analog fashion^19^,^20^. There is also evidence that mature animals, such as monkeys, are able to go beyond representing single magnitudes, and can represent proportional relations between two magnitudes^11^,^21^,^22^. Additionally, Rugani *et al*.^20^ found that day-old chicks can represent proportions of two blocks of continuous area^20^. However, it is unclear whether this same early-developing propensity would be found for discrete stimuli, over which area statistics must be extracted from the bounded objects found in the scene. For purposes of foraging, overall area of a stimulus is the most critical cue to abundance and opportunity, and animals prioritize area when choosing their behavioral response^23^. Thus, chicks may be only able to reason proportionally in an ecologically valid scenario of apparent area discrimination. If chicks - an animal model that is known to master numerical discrimination, ordinality, and arithmetic calculations^15^,^16^,^24^,^25^ - possess a proportional reasoning system that can take as input any type of quantity (either spatial and continuous, or bounded and discrete), then we would expect them to display sensitivity to proportions like the mature animals studied by Vallentin and Nieder^11^ and Drucker *et al*.^22^.

In the current study, we performed two experiments. In Experiment 1, we replicate and extend Rugani *et al*.^20^ by examining whether chicks can selectively respond to, and then generalize from, a particular proportion of two types of discrete object arrays. In a training session, four-day-old domestic chicks were rewarded with food after circumnavigating a panel depicting a target proportion of discrete, bounded objects (4 green dots for every 1 red dot, or 4:1; see Table 1), which was presented side-by-side with a neutral, unrewarded, proportion of 2:1. In a set of subsequent testing trials chicks were presented with new (never seen during training) sets of 2:1 and 4:1 exemplars, and their first panel circumnavigated was scored. If chicks can represent proportions over discrete objects and not simply continuous area (see Table 2), they will preferentially navigate to the previously rewarded proportion during testing trials. In Experiment 2 we investigated whether day-old chicks could identify a specific ratio and discriminate it from a smaller and a larger ratio. Chicks learned to respond to stimuli characterized by a 2:1 proportion, specified by green and red dots (positive stimulus) when compared either with stimuli depicting a smaller (1:1) or a larger (4:1) proportion (neutral stimuli). As in Experiment 1, we created two sets of stimuli, one for training and one for test (see Table 3), which controlled for absolute overall number or number of each type of sub-element for each set and for each ratio (see Table 4). During training and testing a positive stimulus was always coupled to a neutral stimulus (4:1 or 1:1). If chicks are able to form an absolute, and not relative, representation of a particular proportion, they will preferentially navigate to new examples of the previously rewarded target proportion when paired with new examples of a neutral stimulus that depicts either a larger or smaller proportion.

## Results

**Figure 1:**
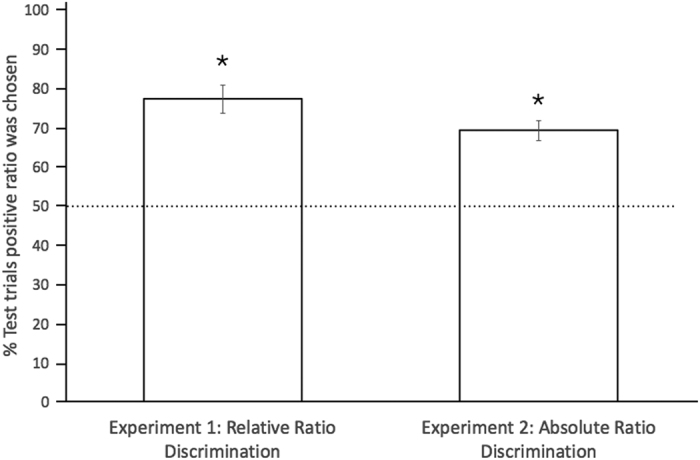
Performance by subjects in both experiments. Error bars indicate +/− one SEM. The dotted line indicates the chance responding level. Asterisks indicate a significant difference from chance, using one-sample t-tests against. 50 with an alpha level of p < 0.05 (See also Supplementary Information).

Chicks can extract a proportional value and productively use it in new contexts. Moreover, they can generalize to new exemplars and new numbers. Results of both experiments are depicted in Fig. 1. On each test trial, we scored the first panel circumnavigated by the chicks. We calculated the number of correct trials and the percentages were computed as: (Number of Correct Choices/20) ×100. In Experiment 1 chicks (n = 10) circumnavigated the panel depicting the positive stimulus (4:1) at above-chance levels: M = 77.18%, SE = 3.53; one-sample t-tests against 50% chance value t(9) = 7.71, p < 0.001. In order to assess whether the overall performance depended on learning, which might have occurred during testing, we considered the first five trials of each session. From the first trials chicks’ performance was statistically above chance (M = 80.00%, SE = 4.22, one-sample t-tests against 50% chance value t(9) = 7.12, p < 0.001), indicating that they could abstract ratios and generalize them to new exemplars immediately after training. In a further analysis we selectively considered the very first trial, and computed the number of chicks that chose the correct proportion; as a group, chicks significantly selected the correct proportion (10/10, binomial sign test p < 0.01).

In Experiment 2, a paired-sample t-test did not reveal any difference between the neutral stimulus types (4:1 : M = 70.00, SE = 3.69 and 1:1: M = 68.33, SE = 3.86; t(10) = 0.30; p = 0.77). Therefore we merged the two stimuli types, and found that chicks (n = 12) circumnavigated the panel depicting the positive stimulus (2:1 green:red) at above-chance levels: M = 69.17%, SE = 2.53, one-sample t-tests against 50% chance value t (11) = 6.98, p < 0.001. When considering the first five test trials chicks’ performance was statistically above chance (M = 68.33%, SE = 4.58, one-sample t-tests against 50% chance value t(11) = 4.01, p = 0.002). On the very first trial, computing the number of chicks that chose the correct proportion, 9/12 subjects succeeded (binomial test, p = 0.15) (see Supplementary Information for details on statistical analyses).

## Discussion

We investigated the ability of four-day old domestic chicks to extract the proportional relation of two arrays of discrete objects. In Experiment 1, chicks discriminated between two specific proportions, 4:1 *vs.* 2:1, in a task similar to those used to test relative numerical magnitude discrimination. Often animals are trained to respond to a target number (e.g. 4) when it is contrasted with another one (e.g. 8), a task that requires discrimination of a smaller stimulus from a larger one. A different ability is required to solve a number identification task, in which a specific number (e.g. 4) must be chosen over a smaller (e.g. 2) or a larger one (e.g. 8). In this case, successful behavior requires a more abstract identification of an *absolute* quantity, and not only a smaller *vs*. larger discrimination. Thus, in Experiment 2, we trained chicks to respond to a target proportion (2:1), that was paired in half of the training trials with a smaller proportion (1:1) and in the other half with a larger one (4:1). Chicks succeeded in the proportional identification, highlighting their ability to represent the abstracted ratio in an *absolute* manner. Again, chicks at test generalized to new ratios, abstracting the positive proportion from new ratios – specified by discrete numerical magnitudes of red and green dots never seen before – and avoiding multiple different types of irrelevant, neutral ratios. Overall, the present study is the first to demonstrate that, soon after birth, animals represent abstract proportional information from discrete elements and use this information productively in order to guide adaptive behavior.

These results are consistent with those found when testing six-month-old infants on their ability to represent proportions^10^. Infants were habituated with multiple examples of a certain proportion of yellow and blue dots. When presented with the same proportion and a new proportion at test, they looked longer at the new one. While the present study is the first to show the capability of newborn animals to extract proportionality from discrete numbers of items, past studies have found that non-human animals can perceive relational quantity in addition to perceptual features such as overall area. In one such instance, a chimpanzee was able to appreciate fractional equivalence (1/4, 1/2, 3/4, 1) by matching pictures of three kinds of objects – spherical food items, circular wood disks and cylindrical water containers – on the basis of their proportional nature^26^. The animal could match proportions on objects between classes, indicating that this matching went beyond physical resemblances into more abstract proportional comparison. Adult rhesus monkeys are able to match length proportions (1/4, 2/4, 3/4 and 4/4) of bars, irrespective of the bars’ absolute lengths^11^,^21^. Monkeys can learn to discriminate between two arrays depicting different ratios of positive to negative elements (white squares and black dots), regardless of the absolute number of elements in the two arrays^22^.

In a related recent study, domestic chicks preferentially attended to the proportion of different-colored areas, neglecting other information such as the prevalent color or the absolute amount of it^20^. In the current study, the chicks were presented with bound, discrete stimuli and not the pixelated continuous area used in Rugani *et al*.^20^. However, it is an open question as to whether the ratios being capitalized upon for the current study were extracted over numerical magnitudes per se, or spatial extent variables such as area or perimeter (which varied alongside number in the current design). Even if one was able to perfectly control for the use of spatial extent within a ratio-specific number design, recent work casts doubt on whether this is a meaningful dissociation in a proportional context. The representation of one type of ratio (e.g., symbolic, or numeric) evokes the formation of an abstract proportional relation that transcends both symbolic - non-symbolic format^27^ and discrete - continuous format^28^. Thus, the central value in this set of experiments comes not from an emphasis on number proper as a cognitive construct, but rather the fact that these very young animals can precociously represent and discriminate proportional information over discrete sets, and do so in a way that suggests the formation of an absolute, non-relative sense of proportional magnitude akin to that found by Vallentin and Nieder^11^.

The ability documented here could be advantageous for animals in their natural environment. It is well established that the ability to use numerical information, such as “more-than *vs.* less-than” judgments, constitutes a survival advantage^29^. Detecting the difference between magnitudes, however, is not always sufficient to maximize behavior. In some circumstances numerical discrimination *per se* is not informative, and adaptive behaviors require that organisms relate set sizes to each other in the form of proportional comparison. Take, for example, foraging situations. An important concept when foraging is *rate of return*: how successful a foraging attempt will be given the time invested in tandem with the relative yield of one type of desirable food, amongst the number of other competitors or undesirable food. This consideration is inherently proportional. In one such example of this, different numbers of pieces of bread thrown at different rates were simultaneously offered at opposite sides of a lake to free-living mallards; these birds divided themselves between resource patches, as if they were simultaneously considering the amount of food and the number of animals feeding at the two sites^30^. This behavior reflects the capability to understand the relative ratios at each site of the available food to the number of competitors in a naturalistic situation.

Previous comparative studies focusing on numerical understanding have found that the facility to discriminate between two magnitudes is ratio dependent: as the ratio between the numbers to be discriminated becomes larger response times decrease and accuracy increases^31^. This pattern suggests that the responsible cognitive mechanism is the approximate magnitude estimation system, which allows non-verbal representation of quantities and numerical evaluation^8^. The similar performance, both qualitatively and quantitatively, in individuals of different species, including human adults when prevented from using language, suggests that this ancient, non-verbal numerical mechanism is shared across species^6^. The ratio signature also characterizes some limits found in proportional reasoning. For instance, six-month old infants can discriminate 2:1 from 4:1, but not 2:1 from 3:1^10^, a limit that is also present in a numerical discrimination task for infants of the same age^32^,^33^. Moreover, monkeys and humans exhibit similar behaviors when discriminating between different sets of line lengths; discrimination in both species is related to the ratio between the two bar lengths, so that as the proportional difference between the length increases, so does performance^11^. This suggests that, as for whole numbers, the discrimination of proportions depends on the functioning of the approximate magnitude estimation system^11^,^21^, and that organisms possess a sense of absolute proportion in the same way they possess a sense of absolute magnitude. This accords nicely with the results obtained in Experiment 2, in which the chicks successfully went beyond more-than-less-than relative judgments, and were able to select the correct proportion against both smaller and larger ratios, indicating a sense of absolute and not relative proportion. However, the chicks took longer to reach training criteria for Experiment 2 (in which they had to choose 2:1 in the context of 4:1 or 1:1, alternately) compared to Experiment 1 (in which they were trained to choose 4:1 in the context of only one other ratio, 2:1.), 101 trials to 63 trials respectively (see Methods). The formation of this absolute proportion appeared to be more effortful for the chicks, a phenomenon that could readily be tested using this design in other non-verbal populations.

Although the ability to represent ratios over discrete items has been found in other non-human animals (such as rhesus macaques:^22^), these populations often have significant life experience to support this capacity. The present study, on the other hand, shows that the ability to discriminate proportional information is precociously available soon after birth in non-human animals, just as in humans^10^. This multi-faceted process should be considered part of the suite of untrained, early-developing abilities supported by the approximate magnitude system alongside numerical discrimination, simple arithmetic, and ordinal discrimination. In fact, there is evidence that this proportional sensitivity may serve as a useful foundation for advanced mathematical skills, even more so than simple representation and discrimination. Matthews, Lewis, and Hubbard^34^ recently found that individual differences in sensitivity to non-symbolic ratios predict formal math performance in adults, and Mohring, Newcombe, Levine, and Frick^35^ found that children’s ability to reason about non-symbolic proportions was related to their understanding of symbolic fractions. By exploring the phylogenetic development of this capacity in special populations, we can provide unique input to the study of its ontogenetic formation in the human species.

## Methods

### Subjects

Twenty-two “Hybro” domestic chicks took part in this experiment (*Gallus gallus*; N = 10 in Experiment 1, N = 12 in Experiment 2). Chicks were obtained several hours after hatching, from a local commercial hatchery (Agricola Berica, Montegalda, Vicenza, Italy). On arrival, the chicks were housed individually in metal cages (28 × 32 × 40 cm). Water and food were available freely. Chicks were offered mealworms (*Tenebrio molitor* larvae) twice a day, to familiarize them with this reinforcement food. Two hours before training the food was removed from the cages. At the end of training chicks were placed back in their home cages and, two hours later, they underwent testing individually.

### Apparatus

**Figure 2:**
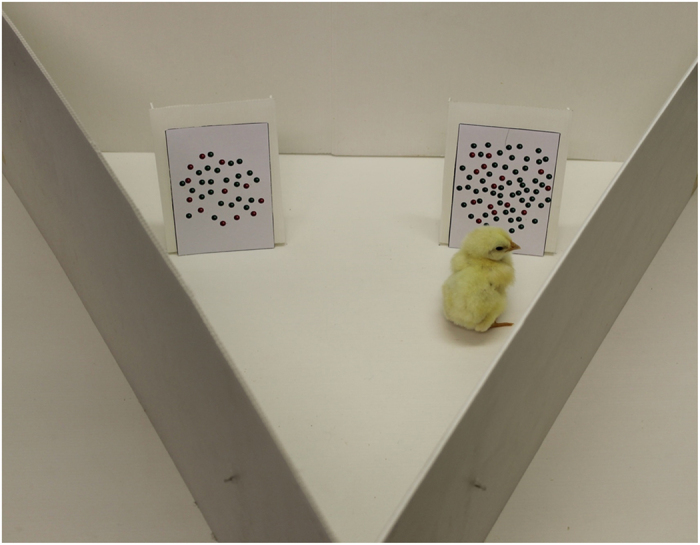
The apparatus used in Experiments 1 and 2. Both panels were present in the apparatus during the training and testing sessions.

The experimental apparatus for both experiments consisted of a triangular arena (width 60 cm, height 20 cm) made of uniformly white plastic panels and flooring (see Fig. 2). A ‘starting’ area was positioned 10 cm from one vertex of the arena. This was delimited by a transparent removable partition (10 cm × 20 cm). The transparent partition was used to confine subjects for five seconds before the beginning of each trial, in order to give them the possibility of seeing the inner apparatus and the stimuli. During training and testing, two identical white plastic panels (16.0 × 8.0 cm) were inside the arena. These were located symmetrically with respect to the starting area, spaced 6.0 cm apart and located 30.0 cm away from the transparent partition. Panels were provided with 3.0 cm sides bent back to prevent chicks from looking behind the panel where the mealworm reinforcement was hidden. During the inter-trial period chicks were moved in a separate box (20 cm × 40 cm × 40 cm) for approximately 30 seconds, to prevent them from seeing the experimenter changing the test stimuli.

### Stimuli

For both experiments, the stimuli were composed of green and red dots designed to appear three-dimensional, generated in Adobe Ilustrator. The diameter of each dot was 0.5 cm. In Experiment 1, the mean number of overall dots during training for the positive stimuli was 30 (24 green and 6 red) and for the neutral stimuli 36 (24 green and 12 red). To ensure that chicks were not responding to the overall number of dots during test, or the mean number of dots for each subelement, the test values were chosen to be psychologically equidistant from those values rewarded in training, with a mean of 8 red dots for both the neutral and positive stimuli (a Weber ratio of 1.33, 8 testing : 6 training) and a mean of 16 and 32 green dots for the neutral and positive stimuli (similar Weber ratios of 1.5 and 1.33, respectively). The mean overall number of dots during testing was 24 and 40 for the neutral and positive stimuli (similar Weber ratios of 1.25 and 1.33 compared to overall number of dots rewarded during training).

In Experiment 2, the mean number of overall dots rewarded in training for the positive stimuli was 24, and the mean numbers of overall dots for the neutral stimuli sets were 48 for the 1:1 stimuli, 30 for the 4:1 stimuli, and 36 for the 2:1 positive stimuli (Weber ratios of 2:1, 1.25, and 1.5). Note that if chicks are going to the overall number of dots, they should approach one of the neutral sets (the 4:1 stimuli) more often and the other neutral set (1:1) less often, in effect cancelling each other out. For the subelements, chicks during training on the positive stimuli were shown on average 16 green dots and 8 red dots. At the neutral 1:1 test, chicks saw an average of 24 green and 24 red dots; at the positive 2:1 test, chicks saw an average of 24 green dots and 12 red dots; at the neutral 4:1 test chicks saw an average of 24 green dots and 6 red dots. For the red dots, this yields Weber discrimination values of 3.0, 1.5, and 1.3. Again, note that if chicks are merely choosing a subelement average that is closest to that trained, the two neutral ratios provide conflicting information as to success of this strategy. For the green dots, this yields identical Weber ratio discrimination values of 1.5 for all stimuli types (1:1, 2:1, and 4:1). To avoid any configurational learning, for each exemplar ratio (e.g., 32:8) we created five different spatial configurations of the dots, yielding 20 pairs total of stimuli.

### Training

Training occurred in the morning of the fourth day. The two panels were in place, with a mealworm located between the starting area and the panel depicting the positive stimulus. The chick was at first placed within the arena, in the starting area, for two minutes, free to move around and to get acquainted with the novel environment (no partition was used to confine the bird in this experimental phase). Five mealworms were subsequently offered to the subject, whilst in the arena, to get it used to feeding in this new environment. Following this acclimation period, the subject underwent the training procedure. Initially a piece of mealworm was positioned in view in front of the panel depicting the positive proportion. Thereafter, the food reinforcement was progressively moved behind the panel depicting the positive ratio, requiring the bird to go behind the panel to retrieve it. For both the training and the testing trials, the chick was placed for approximately 30 seconds between trials in an opaque plastic container located outside of the arena while the experimenter changed the stimuli. The experimenter was located in a laterally neutral location behind the chick – opposite the chick’s line of sight to the panels - throughout the trials to prevent social cueing. The end of the trial was the first circumnavigation of one of the test panels, as determined by the experimenter.

In all correct training trials chicks received reinforcement. To avoid any spatial learning during training, and also during testing, we changed the left–right (L–R) position of the stimulus associated with food/side of presentation of the positive stimulus, following a semi-random sequence (e.g. L–R–L–R–L–L–R–R–L–R–L–R–L–R–L–L–R–R–L–R^36^,^37^. Learning criterion was set at 16/20 trials correct. The training procedure was identical for Experiments 1 and 2. The mean number of trials to reach the learning criterion was 63.10 ± 7.64 in Experiment 1 and 101.25 ± 16.39 in Experiment 2.

### Test

Two hours after the end of training each chick underwent the test, which consisted of 20 trials. At the beginning of each trial, the chick was confined to the starting area, behind the transparent partition, from where it could see the two panels. The stimuli were located on the front part of each panel (facing the starting area). In each trial, a pair of test stimuli was used and the left–right position of the positive stimulus was changed from trial to trial according to the semi-random sequence described above. The chick remained confined in the starting area for 5 seconds; afterwards, the transparent partition was removed and the chick was free to move. When the chick had placed its head and about ¾ of its body behind a panel it was deemed to have made a choice, at which point the trial was considered to be over (only the first panel chosen was taken into consideration). If the first panel approached corresponded to the one depicting the proportion associated at training with food the response was considered as ‘correct’, otherwise it was considered ‘incorrect’.

The chicks’ behavior was observed from a monitor connected to a video camera so as not to disturb the chicks by direct observation. All trials were video-recorded. The chicks’ performance was scored off-line by a coder naïve to the hypotheses of the study. We calculated the number of correct trials and the percentages were computed as: (Number of Correct Choices/20) ×100. To ensure no item effects were influencing the results, the training and testing sets were switched for half the chicks.

In Experiment 1, testing consisted in 20 consecutive trials. In each trial a new pair of stimuli of the new set was used. During testing the food reinforcement was available behind the panel depicting the positive ratio only in some pre-established trials (trial numbers 4, 5, 7, 10, 13, 14, 16 and 19^20^), and chicks could gain the food only by choosing correctly in those trials. All other trials were unrewarded.

In Experiment 2, to better control for any effect of learning during testing, we changed the testing procedure. The test phase consisted of two testing sessions, each of which was composed of 30 trials: 20 training trials (tr) and 10 testing trials (T) mixed together (tr, tr, T, tr, tr, tr, T, tr, T, tr, tr, T, tr, tr, T, tr, tr, tr, T, tr, tr, T, tr, T, tr, T, tr, tr, tr, T). In training trials we used the set of stimuli used at training, and chicks, giving a correct response, received the reward. In testing trials we used the second set of stimuli and chicks never received the reward.

### Ethics Statement

The experiments complied with all applicable national and European laws concerning the use of animals in research and were approved by the Italian Ministry of Health (permit number: 32662 emitted on 10/1/2012).

All procedures employed in the experiments included in this study were examined and approved by the Ethical Committee of the University of Padua (Comitato Etico di Ateneo per la Sperimentazione Animale –C.E.A.S.A.) as well as by the Italian National Institute of Health (N.I.H).

## Additional Information

**How to cite this article**: Rugani, R. *et al*. Ratio abstraction over discrete magnitudes by newly hatched domestic chicks (*Gallus gallus*). *Sci. Rep.* **6**, 30114; doi: 10.1038/srep30114 (2016).

## Supplementary Material

Supplementary Information

## Figures and Tables

**Table 1:** Experiment 1: Generalization of Trained Ratio.

Proportion	Neutral Stimuli (2:1)		Positive Stimuli (4:1)		Total number of dots in 2:1 vs. 4:1 stimuli
Color of dots	Number of green dots	Number of red dots	Number of green dots	Number of red dots	
Training Stimuli					
Ratio comparisons	12	6	32	8	18 vs. 40
	28	14	12	3	42 vs. 15
	20	10	44	11	30 vs. 55
	36	18	8	2	54 vs. 10
*Mean*	*24*	*12*	*24*	*6*	
Testing Stimuli					
Ratio comparisons	24	12	56	14	36 vs. 70
	4	2	36	9	6 vs. 45
	22	11	16	4	33 vs. 20
	14	7	20	5	21 vs. 25
*Mean*	*16*	*8*	*32*	*8*	

**Table 2:** Number of red, green and total dots, and the relative overall area and overall perimeter of each stimulus used in Experiment 1.

Proportions	Neutral Stimuli (2:1)									Positive Stimuli (4:1)								
	Number (N) Area (A) and Perimeter (P) of green dots			Number (N) Area (A) and Perimeter (P) of red dots			Total Number (N) Area (A) and Perimeter (P) of dots			Number (N) Area (A) and Perimeter (P) of green dots			Number (N) Area (A) and Perimeter (P) of red dots			Total Number (N) Area (A) and Perimeter (P) of dots		
	N	A	P	N	A	P	N	A	P	N	A	P	N	A	P	N	A	P
Training Stimuli																		
Values (N, A and P) for each ratio comparison	12	9.48	37.68	6	4.74	18.84	18	14.22	56.52	32	25.28	100.5	8	6.32	25.12	40	31.6	125.6
	28	22.12	87.92	14	11.06	43.96	42	33.18	131.9	12	9.48	37.68	3	2.37	9.42	15	11.85	47.1
	20	15.8	62.8	10	7.9	31.4	30	23.7	94.2	44	34.76	138.2	11	8.69	34.54	55	43.45	172.7
	36	28.44	113	18	14.22	56.52	54	42.66	169.6	8	6.32	25.12	2	1.58	6.28	10	7.9	31.4
*Mean*	*24*	*18.96*	*75.36*	*12*	*9.48*	*37.68*				*24*	*18.96*	*75.36*	*6*	*4.74*	*18.84*			
Testing Stimuli																		
Values (N, A and P) for each ratio comparison	24	18.96	75.36	12	9.48	37.68	36	28.44	113	56	44.24	175.8	14	11.06	43.96	70	55.3	219.8
	4	3.16	12.56	2	1.58	6.28	6	4.74	18.84	36	28.44	113	9	7.11	28.26	45	35.55	141.3
	22	17.38	69.08	11	8.69	34.54	33	26.07	103.6	16	12.64	50.24	4	3.16	12.56	20	3.16	12.56
	14	11.06	43.96	7	5.53	21.98	21	16.59	65.94	20	15.8	62.8	5	3.95	15.7	25	19.75	78.5
*Mean*	*16*	*12.64*	*50.24*	*8*	*6.32*	*25.12*				*32*		*100.5*	*8*	*6.32*	*25.12*			

**Table 3:** Experiment 2: Simultaneous Discrimination of Trained Ratio From Larger and Smaller Ratios.

Proportions	Neutral Stimuli (1:1)			Positive Stimuli (2:1)			Neutral Stimuli (4:1)		
	Number of green dots	Number of red dots	Total number of dots	Number of green dots	Number of red dots	Total number of dots	Number of green dots	Number of red dots	Total number of dots
Training Stimuli									
Ratio comparisons	22	22	44	12	6	18	12	3	15
	8	8	16	18	9	27	16	4	20
	4	4	8	14	7	21	36	9	45
	30	30	60	20	10	30	64	16	80
*Mean*	*16*	*16*		*16*	*8*		*32*	*8*	
Testing Stimuli									
Ratio comparisons	36	36	72	8	4	12	24	6	30
	12	12	24	28	14	42	12	3	15
	7	7	14	20	10	30	52	13	65
	41	41	82	40	20	60	8	2	10
*Mean*	*24*	*24*		*24*	*12*		*24*	*6*	

**Table 4:** Number of red and green dots and the relative overall area and overall perimeter of each stimulus used in Experiment 2.

Proportions	Neutral Stimuli (1:1)									Positive Stimuli (2:1)									Neutral Stimuli (4:1)								
	Number (N) Area (A) and Perimeter (P) of green dots			Number (N) Area (A) and Perimeter (P) of red dots			Total Number (N) Area (A) and Perimeter (P) of dots			Number (N) Area (A) and Perimeter (P) of green dots			Number (N) Area (A) and Perimeter (P) of red dots			Total Number (N) Area (A) and Perimeter (P) of dots			Number (N) Area (A) and Perimeter (P) of green dots			Number (N) Area (A) and Perimeter (P) of red dots			Total Number (N) Area (A) and Perimeter (P) of dots		
	N	A	P	N	A	P	N	A	P	N	A	P	N	A	P	N	A	P	N	A	P	N	A	P	N	A	P
Training Stimuli																											
Values (N, A and P) for each ratio comparison	22	17.38	69.08	22	17.38	69.08	44	34.76	138.2	12	9.48	37.68	6	4.74	18.84	18	14.22	56.52	12	9.48	37.68	3	2.37	9.42	15	11.85	47.1
	8	6.32	25.12	8	6.32	25.12	16	12.64	50.24	18	14.22	56.52	9	7.11	28.26	27	21.33	84.78	16	12.64	50.24	4	3.16	12.56	20	15.8	62.8
	4	3.16	12.56	4	3.16	12.56	8	6.32	25.12	14	11.06	43.96	7	5.53	21.98	21	16.59	65.94	36	28.44	113	9	7.11	28.26	45	35.55	141.3
	30	23.7	94.2	30	23.7	94.2	60	47.4	188.4	20	15.08	62.8	10	7.9	31.4	30	23.7	94.2	64	50.56	201	16	12.64	50.24	80	63.2	251.2
*Mean*	*16*	*12.64*	*50.24*	*16*	*12.64*	*50.24*				*16*	*12.64*	*50.24*	*8*	*6.32*	*25.12*				*32*	*25.28*	*100.5*	*8*	*6.32*	*25.12*			
Testing Stimuli																											
Values (N, A and P) for each ratio comparison	36	28.44	113	36	28.44	113	72	56.88	226.1	8	6.32	25.12	4	3.16	12.56	12	9.48	37.68	24	18.96	75.36	6	4.74	18.84	30	23.7	94.2
	12	9.48	37.68	12	9.48	37.68	24	18.96	75.36	28	22.12	87.92	14	11.06	43.96	42	33.18	131.2	12	9.48	37.68	3	2.37	9.42	15	11.85	47.1
	7	5.53	21.98	7	5.53	21.98	14	11.06	43.96	20	15.8	62.8	10	7.9	31.4	30	23.7	94.2	52	41.08	163.3	13	10.27	40.82	65	51.35	204.1
	41	32.39	128.7	41	32.39	128.7	82	64.78	257.5	40	31.6	125.6	20	15.08	62.8	60	47.4	188.4	8	6.32	25.12	2	1.58	6.28	10	7.9	31.4
*Mean*	*24*	*18.96*	*75.36*	*24*	*18.96*	*75.36*				*24*	*18.96*	*74.36*	*12*	*9.48*	*37.68*				*24*	*18.96*	*74.36*	*6*	*4.74*	*18.84*			
