# Supplementary material for: Ratio abstraction over discrete magnitudes by newly hatched domestic chicks (Gallus gallus)
Source: Sci Rep. 2016 Jul 28;6:30114. doi: 10.1038/srep30114 (PMC4964567; doi:10.1038/srep30114)
Supplement: Supplementary Information [file srep30114-s1.pdf]

Supplementary Information

Ratio abstraction over discrete magnitudes by newly hatched domestic chicks (*Gallus gallus*)

Rosa Rugani, Koleen McCrink, Maria-Dolores de Hevia, Giorgio Vallortigara, Lucia Regolin

Supplementary Table S1

Subject	Number of training trials to learning criterion	Correct responses (%) in all 20 testing trials	Correct responses (%) in the first 5 trials	First trial (C= correct I= incorrect
1	42	60	60	C
2	53	65	80	C
3	20	80	80	C
4	78	63.158*	80	C
5	75	85	80	C
6	81	90	100	C
7	68	80	100	C
8	104	90	80	C
9	42	73.684*	80	C
10	68	85	60	C

Table S1. Data of Experiment 1. The total number of trials needed during training to reach the learning criterion, the correct number of valid trial in overall test, the number of correct responses emitted by each chick in the first five testing trials and the data of the very first trial were reported. For each chick were computed the percentage of correct responses by dividing the number of correct responses by the total number of valid trial.

* Because the chicks' performance was scored off-line by a coder naïve to the hypotheses of the study, for subjects number 4 and 9 one testing trail was not clearly detectable on the videos and therefore for these two subjects total number of valid trials was 19. For these two subjects we calculated the number of correct trials and the percentages were computed as: (Number of Correct Choices / 19) x 100.

Supplementary Table S2.

Subject	Number of training trials to learning criterion	Correct responses (%) in all 20 testing trials	Correct responses (%) in the first 5 trials	First trial (C= correct I= incorrect
1	102	75	80	I
2	186	65	80	I
3	172	70	60	C
4	121	75	60	C
5	49	70	60	C
6	126	80	100	C
7	123	75	80	C
8	20	70	60	C
9	28	60	60	C
10	144	70	80	I
11	114	45	40	C
12	30	70	60	C

Table S1. Data of Experiment 2. The total number of trials needed during training to reach the learning criterion, the correct number of valid trial in overall test, the number of correct responses emitted by each chick in the first five testing trials and the data of the very first trial were reported.
